# Phonon properties of graphene derived from molecular dynamics simulations

**DOI:** 10.1038/srep12923

**Published:** 2015-08-28

**Authors:** Emmanuel N. Koukaras, George Kalosakas, Costas Galiotis, Konstantinos Papagelis

**Affiliations:** 1 Institute of Chemical Engineering Sciences, Foundation of Research and Technology-Hellas (FORTH/ICE-HT), Stadiou Street, Platani, Patras, 26504 Greece; 2 Department of Materials Science, University of Patras, Patras, 26504 Greece; 3 Crete Center for Quantum Complexity and Nanotechnology (CCQCN), Physics Department, University of Crete, 71003 Heraklion, Greece; 4 Department of Chemical Engineering, University of Patras, Patras 26504 Greece

## Abstract

A method that utilises atomic trajectories and velocities from molecular dynamics simulations has been suitably adapted and employed for the implicit calculation of the phonon dispersion curves of graphene. Classical potentials widely used in the literature were employed. Their performance was assessed for each individual phonon branch and the overall phonon dispersion, using available inelastic x-ray scattering data. The method is promising for systems with large scale periodicity, accounts for anharmonic effects and non-bonding interactions with a general environment, and it is applicable under finite temperatures. The temperature dependence of the phonon dispersion curves has been examined with emphasis on the doubly degenerate Raman active Γ-E_2g_ phonon at the zone centre, where experimental results are available. The potentials used show diverse behaviour. The Tersoff-2010 potential exhibits the most systematic and physically sound behaviour in this regard, and gives a first-order temperature coefficient of *χ* = −0.05 cm^−1^/K for the Γ-E_2g_ shift in agreement with reported experimental values.

In crystalline materials the characteristics of phonons are of fundamental importance. Many properties of the materials are directly or indirectly determined from the phonon spectrum and phonon density of states, such as the thermal conductivity, the specific heat, and the thermal expansion^1^. Several computational methods are available for extracting phonon properties of systems of varying dimensionality and complexity, under the general terms of linear response and direct force constant methods^1^,^2^,^3^,^4^,^5^,^6^,^7^,^8^,^9^. A very effective method for deriving phonon dispersion curves and phonon density of states, valid for finite temperatures as opposed to absolute zero, is through molecular dynamics simulations of the modelled material^10^,^11^,^12^,^13^,^14^. This method has certain advantages; anharmonic interactions between atoms are implicitly taken into account, since it is based on processing of the atomic trajectories. Furthermore, this method is promising for systems with large scale periodicity and/or periodically induced defects as it is adaptable for usage with supercells. Moreover, it is straightforward to adopt the method on systems interacting with a substrate or with a more general environment.

For graphitic materials phonons play an important role; analysis of Raman active modes is used to identify characteristics such as the disorder (crystallinity) of the sample, the tension and compression state, the number of graphitic layers, interlayer coupling, oxidation, and more (see Ref. 15 and references therein). As a means to compare the vibrational response of graphene as derived by widely used classical potentials with respect to experiment, we have developed a methodology and implemented an in-house computer code to compute phonon dispersion curves from molecular dynamics simulations. We have suitably adapted the approach presented in Refs 10, 11, 12, 13 to efficiently handle two dimensional materials such as graphene. Previous efforts using this method are on simple systems with only up to three dispersion curves^10^,^13^,^16^,^17^,^18^,^19^,^20^. In this work we successfully apply the above method to investigate the full dispersion curves of graphene. This is not trivial as the spectral resolution required to obtain the six dispersion curves of graphene demands careful treatment in modelling and of various numerical aspects. In this respect we have calculated all the optical and acoustic branches for the high symmetry directions in *k*-space and examine their temperature dependence. Special attention is given to the doubly degenerate Γ-E_2g_ mode of the TO and LO branches at the Γ point of the Brillouin zone (BZ) in order to compare its temperature dependence with existing experimental data. We note that although there are several experimental studies on the temperature dependence, corresponding theoretical investigations in this direction are lacking.

The paper is organised as follows. In the following section we describe in detail the implemented method along with aspects that are taken into consideration specifically applied to graphene. In the next section we report results for the obtained phonon dispersion curves of graphene by employing widely used classical potentials and assess their performance. In the final section we specifically examine the temperature dependence of the Γ-E_2g_ vibrational mode using each of the considered potentials. Then, for the potential that better describes this dependence we present the phonon dispersion curves at different temperatures. Pertinent conclusions of this work are given at the end.

## Method of Computation

The objective is to obtain the oscillation frequencies *ω* that correspond to a given wavevector ***k***. We consider the velocity distribution in reciprocal space (*k*-space) that is obtained by the spatial Fourier transform


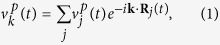


where *j* sums over unit cells (once for each particular atom of the crystal basis, as discussed further below; *R*_*j*_(*t*) are the position vectors of the corresponding atoms) and *p* is a component (*x*, *y*, or *z*) in a Cartesian coordinate system (i.e. 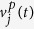 is the *p*-component of the velocity of the atom *j* at position *R*_*j*_(*t*)). Taking the velocity as a wide-sense stationary random process and employing the Wiener-Khintchine theorem the power spectral density (PSD) is given by the spectral decomposition of the *k*-space velocity autocorrelation function (*k*VACF) 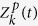 [Ref. 21]


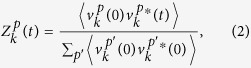


where averages denote time averages (if this is to be considered as an ergodic process). Once the PSD is calculated via the temporal Fourier transform of the *k*VACF, the phonon frequencies corresponding to the wavevector ***k*** are obtained through the position of the peaks in the PSD.

In practice the quantities involved result from molecular dynamics simulations which are carried out with finite time steps. As such, the variables are discrete and the velocity autocorrelation function needs to be calculated as such. The problem is thus reduced to calculating the reciprocal space velocity autocorrelation *sequence* (*k*VACS). We denote 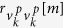 as the numerator of the *k*VACS, where *m* is an integer index denoting time differences from a reference point (instead of the continuous function of time *k*VACF). With 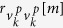 at hand, to calculate the *k*VACS one only needs to divide with the sum over Cartesian components at time index zero, 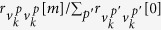. More so, the autocorrelation sequence is not infinite but instead truncated to the total number of steps, *N*, available from the MD simulation. As a result, estimates of the velocity autocorrelation expectation value, 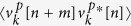



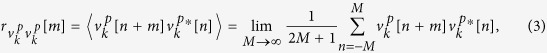


need to be employed^21^, where 2*M* + 1 is the total size of the discreet sequence. The most straightforward line of action is to adjust the previous equation to the available data range of *N* data samples and consider only positive time indices 0 ≤ *m* ≤ *N* − 1, which leads to


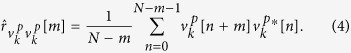


The sequence 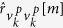 is an unbiased estimator as it leads to unbiased estimates of the true (infinite/non-truncated) sequence. Another autocorrelation estimator, denoted as the biased estimator, for positive time indices is given by 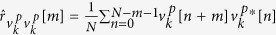.

Both estimators have zero variance for large values of *N*, so they are statistically consistent (properties of these estimators can be found in Ref. 21). Here we use the unbiased estimator, eqn (4). The PSD can be calculated by fast Fourier transformation (FFT) with respect to time of either the biased or unbiased autocorrelation sequence estimators. To avoid large variance for large time indices when using the unbiased estimator, Blackman and Tukey suggest^22^ that the number of indices to be estimated should be about 10% of the available data samples, i.e. *L*_max_ ≈ *N*/10, which however was not needed in our case.

The described method for producing dispersion curves accounts for anharmonic effects as the corresponding information is embedded in the trajectories and velocities of the atoms. While at least one other method^14^ manages to account for anharmonic effects by utilizing molecular dynamics simulations, the *k*VACS method has the advantage that it produces stable results throughout the Brillouin zone, including the region near and at the Γ point.

Since ergodicity is not ensured and the *k*VACS is truncated we considered an ensemble for the *k*VACS, as described below. In our implementation we perform the FFTs of this ensemble averaged *k*VACS using the portable and open source FFTW3 library^23^. The molecular dynamics simulations were performed using the LAMMPS^24^ software package.

**Figure 1:**
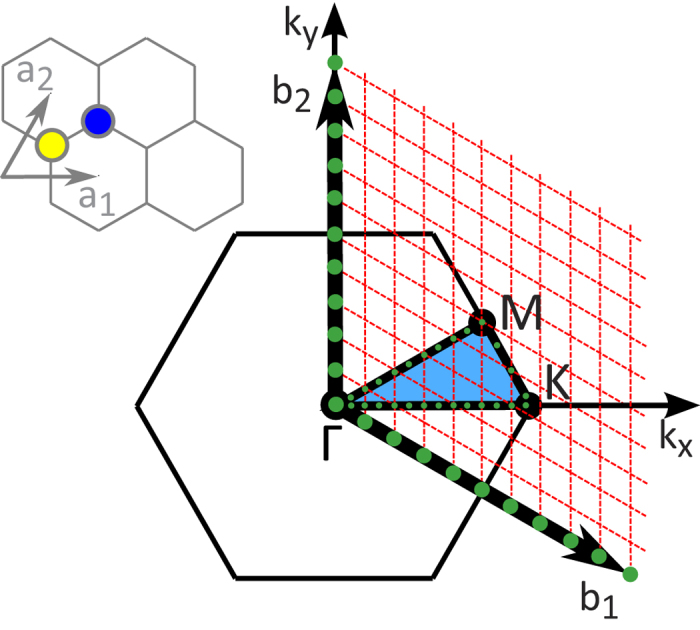
First Brillouin zone of graphene. The orientation of the direct lattice is also shown (not under scale). The yellow and blue circles denote the carbon atoms A and B of the trigonal sublattices, respectively. The reciprocal lattice vectors, **b**_**1**_ and **b**_**2**_, are partitioned as shown by the green dots (for clarity only 10 of the 20 partitioning segments are shown, considering a computational cell of 20 × 20 unit cells). The allowed ***k***-vectors along the high symmetry path ΓKMΓ are shown by the small green dots (in this case all allowed ***k***-vectors are shown, obtained from the full partitioning). The shaded blue area represents the irreducible Brillouin zone.

To obtain a resolution suitable to clearly acquire the six dispersion curves of graphene several technical considerations should be taken into account. (i) A triclinic computation cell should be used as it has the same number of unit cells on each of the rows (or columns); in other words the symmetry of the unit cell is transcribed to the computational cell. This permits for the proper quantization of the Brillouin zone (BZ) and should also be taken into consideration when defining the ***k***-vector path. With orthogonal computation cells the number of permitted ***k***-vectors is ill defined. (ii) Several realisations of each simulation should be taken, from which an averaged *k*VACS should be produced and then Fourier transformed. As expected, the standard deviation of the autocorrelation function is reduced as the number of simulations performed increases. (iii) Double precision variables should be used throughout. This includes the trajectories and velocities taken from the molecular dynamics simulation package. It is also important to use double precision when defining the *k*-path vectors. (iv) Finally, as described above, in order to obtain all branches of the phonon dispersion in the general case the whole procedure, starting from the summation in eqn (1), needs to be performed once for each atom of the crystal basis. Especially for graphene which has two trigonal sublattices A and B with the *same* atom type (see Fig. 1), the autocorrelation sequence calculated using only one sublattice (A or B) can produce all of the dispersion branches.

### Dispersion Curves

Applying the method outlined above, we have calculated the dispersion curves of graphene using the Tersoff^25^,^26^, Tersoff-2010 (a reparameterisation of the original Tersoff potential by Lindsay and Broido^27^, optimised to better represent lattice dynamical properties of graphene), LCBOP^28^ and AIREBO^29^ potentials. The molecular dynamics simulations were performed using a triclinic computational cell of 20 × 20 unit cells (overall 800 carbon atoms) and periodic boundary conditions. The computational cell was initially relaxed for each potential. Randomised velocities (for all three dimensions) were attributed to the atoms, within a Gaussian distribution, corresponding to the desired temperature and an initial equilibration at constant energy (NVE ensemble) was performed. The corresponding lattice parameter was computed by performing a subsequent equilibration within the isothermal–isobaric ensemble (NPT) at zero pressure and the desired temperature each time. The obtained lattice parameter was used in defining the BZ edges in each case. The trajectories and velocities needed for the computation of the dispersion curves were produced from the final round of simulations within the microcanonical ensemble (NVE) using the lattice parameters obtained from the previous rounds. A very fine time step of 0.05fs was used and the trajectory and velocities were saved every 10 time steps. The final NVE simulations lasted for 655360 time steps each of which corresponds to about 32.8 ps. To produce more reliable statistics, multiple NVE realizations (in a total of 10 for each case) were performed, and the average *k*-space velocity autocorrelation sequence was Fourier transformed. Between these realizations a velocity rescaling was applied corresponding to the given temperature followed by an NPT equilibration.

For a *n* × *n* triclinic computational cell the allowed ***k***-vectors are obtained through a grid derived by partitioning each of the reciprocal lattice vectors, ***b***_1_ and ***b***_2_, into *n* segments. For *k*-points other than those mentioned above the PSD exhibits factitious peaks. With a lattice orientation such that one direct lattice vector (say ***a***_1_) is along the *x*-axis, as shown in Fig. 1, the general form of the allowed ***k***-vectors is


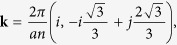


where *i*, *j* count sampling sections along the ***b***_1_- and ***b***_2_-directions respectively, and *a* is the lattice constant. It is straightforward to show that the allowed ***k***-vectors along the directions connecting the high-symmetry points, Γ, K and M are obtained as follows: i) along **ΓK** the allowed ***k***-vectors have *i* = 2*j* and *i* ≤ 2*n*/3 (*i*_max_ = 13 for *n* = 20), ii) along **ΓM** they have *i* = *j* and *i* ≤ *n*/2 (*i*_max_ = 10 for *n* = 20), and iii) along **MK** they have *i* = −*j* + *n* with *n*/2 ≤ *i* ≤ 2*n*/3 (for *n* = 20 this becomes *i*_min_ = 10 and *i*_max_ = 13).

**Figure 2:**
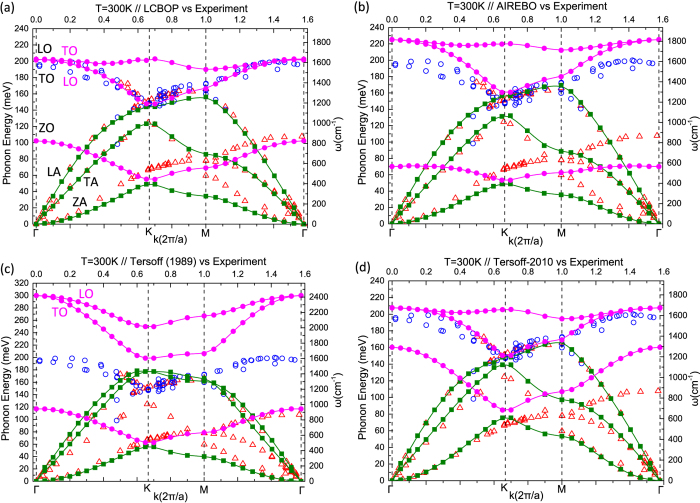
Phonon dispersion curves of graphene calculated using (**a**) the LCBOP, (**b**) the AIREBO, (**c**) the original Tersoff (1989), and (**d**) the reparameterised Tersoff-2010 potential, at *T* = 300 K. The reported MSE, MAE and RMSD values in Table 1 were obtained using these dispersion curves. Solid circles and squares correspond to numerical results of optical and acoustic branches, respectively. Open symbols correspond to experimental data taken from Refs 30,31.

In Fig. 2(a–d) we show the results of our calculations obtained for each of the potentials under consideration (Tersoff, Tersoff-2010, LCBOP, and AIREBO). The dispersion curves were calculated at a temperature of *T* = 300 K to facilitate the comparison with the experimental values which were measured at room temperature^30^,^31^. In the Supplementary Information we compare the results derived through the presented method at low temperatures (*T* = 60 K) with the corresponding ones obtained by a direct diagonalization of the dynamical matrix for the Tersoff and Tersoff-2010 potentials. Excellent agreement is obtained, demonstrating the accuracy of the proposed method. In Fig. 2 the calculated results are given in solid circles for the optical modes and solid squares for the acoustic modes. Hollow symbols represent experimental data from Maultzsch *et al.*^30^ and Mohr *et al.*^31^, which we have included for comparison. As can be seen, each of the potentials succeeds on producing a good description of some branches, but none manages to do so for all branches. The best overall description is seemingly provided by LCBOP. In Table 1 we list for each potential the mean signed error (MSE), mean absolute error (MAE) and the root mean square deviation (RMSD) for each branch separately and also for the overall phonon dispersions, compared to the corresponding experimental values (the formulas used are provided in the Supplementary Information). The calculated frequencies used were taken at the *k* value of each experimental data point by interpolation between two sampling points in the specific *k*-region. Along with the original Tersoff potential, that properly describes the ZO branch, LCBOP also provides an acceptable description of the ZO branch. However, the Tersoff potential fails dramatically on the other two optical modes, while LCBOP provides rather accurate TO and LO branches, arguably the most accurate around the Γ point. A significant improvement in the description of the TO and LO branches is obtained by the reparameterisation of the Tersoff potential by Lindsay and Broido^27^. However, this is at the cost of a much worse description of the ZO branch (as compared to the original Tersoff potential). The AIREBO and Tersoff-2010 compete in accuracy depending on the specific branch. The AIREBO potential overestimates the TO and LO branches (especially around Γ) somewhat more than the Tersoff-2010 potential. Also, while AIREBO underestimates the ZO branch, Tersoff-2010 overestimates it. The description of the acoustic branches is more or less on equal footing, with the AIREBO providing a more accurate TA branch and Tersoff-2010 providing the most accurate ZA branch (of all the given potentials). What is very apparent in the dispersion curves from all of the potentials used here is their general failure in describing the highest optical mode around the K point.

A feature that is lacking in all of the calculated dispersion curves is the presence of discontinuities of the derivative at the K and Γ points. The discontinuities in the highest optical branches at K and Γ that are apparent in the corresponding experimental data shown in Fig. 2 signify the occurrence of Kohn anomalies^30^,^31^,^32^,^33^,^34^,^35^. These appear at high symmetry points when the screening of atomic vibrations from conduction electrons changes rapidly^32^,^34^,^35^. The cusps cannot be described by the classical potentials employed here for which the highest optical branches near K and Γ have a flat slope^34^,^35^. However, since the Kohn anomaly shape is expected to be temperature independent^36^, the potentials can in principle capture the correct (within the accuracy of any given potential) temperature dependence at the high symmetry points. We explicitly denote the branches in Fig. 2(a,c) in black for the assignments by means of experiment and *ab initio* calculations^30^,^31^ and in magenta for our calculations. We note that the highest optical branch produced by the LCBOP, AIREBO, and Tersoff-2010 potentials is TO since they are incapable of capturing the expected softening near the K point as a result of the Kohn anomaly. An exception is the original Tersoff potential for which the highest optical branch is LO, but as we mentioned fails dramatically on the highest optical branches regardless.

### Temperature Dependence

In all sp^2^ hybridized carbon based materials the Raman spectrum exhibits the *G* peak that corresponds to an in-plane phonon with E_2g_ symmetry (i.e. doubly degenerate). Very few experimental results are available on the temperature dependence of the *G* peak frequency for carbon nanotubes and even fewer for graphene, which however provide the scale of the dependence. A linear softening, *ω* = *ω*_0_ + *χT* (*ω*_0_ is the extrapolated *G* peak frequency at zero temperature), of the Γ-E_2g_ mode is noted, which is characterized by the first-order temperature coefficient, *χ*. Calizo *et al.*^37^ measured the temperature dependence of the *G* peak of monolayer graphene for a wide temperature range from 83 to 373 K and estimated the first-order temperature coefficient *χ* = −0.0162 cm^−1^/K. A similar softening of the Γ-E_2g_ frequency was observed^38^ for graphene covering 20 nm depressions for changes in temperature from 4.2 K to 77 K and 300 K. More recent results^39^ for graphene over SiO_2_ substrate measured in a temperature range of 298 K to 560 K give a convergent value (over heating cycles) of *χ* = −0.03 cm^−1^/K, and an estimated intrinsic value of *χ* = −0.02 cm^−1^/K. Moreover, there are several experimental works on the temperature dependence of the G peak of carbon nanotubes keeping in mind the peculiarities of carbon nanotubes compared to graphene such as curvature and electron confinement effects^36^,^40^. Measurements performed on electrically heated single-walled suspended carbon nanotubes by Deshpande *et al.*^41^ revealed a softening of the *G*^+^ and *G*^−^ peaks (curvature splits the graphene G mode into two components, one parallel, G^+^, and one perpendicular, G^–^, to the tube axis) which was related linearly to temperature, with almost the same first-order temperature coefficient for both peaks around *χ* = −0.03 cm^−1^/K. Similar results were found by Zhang *et al.*^42^ for single-walled carbon nanotubes with a wide range of diameters. They reported that the diameter of the nanotubes had no obvious influence on the temperature dependence of the *G* mode and calculated an average value of *χ* = −0.026 cm^−1^/K (over nanotubes of different diameters). Fine measurements on single-walled carbon nanotubes^43^ within the temperature range of 240–600 K reveal a subtle quadratic temperature dependence of *G*^+^ and *G*^−^ band redshifts rather than a linear one. Nevertheless, the average first-order temperature coefficients for the *G*^+^ and *G*^−^ peaks are about *χ* = –0.026 cm^−1^/K.

**Figure 3:**
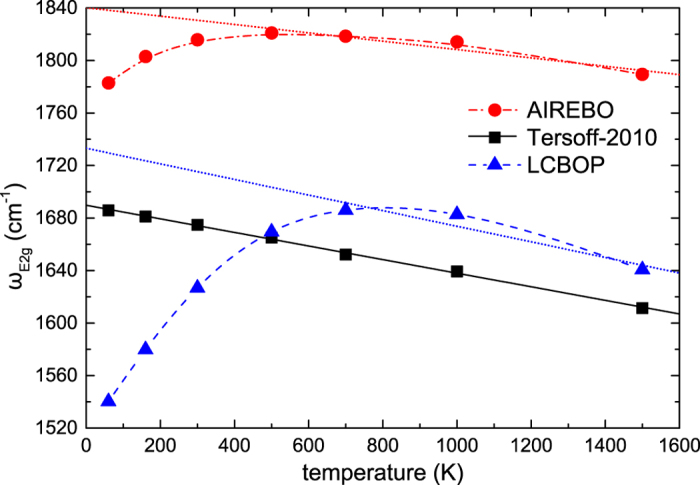
Temperature dependence of the Γ-E_2g_ phonon frequency in graphene for the AIREBO (red circles), Tersoff-2010 (black squares), and LCBOP (blue triangles) potentials. The solid line is a linear fit of the data for the Tersoff-2010. Dashed lines are guides to the eye for the AIREBO and LCBOP data. Dotted lines are linear fits to data points at *T* > 500 K for AIREBO and *T* > 700 K for LCBOP.

We opted to examine the temperature dependence of the Γ-E_2g_ mode of graphene using the AIREBO, Tersoff-2010 and LCBOP potentials. The original Tersoff was not included since it grossly overshoots the frequency of the mode, as shown in Fig. 2c. The Γ-E_2g_ is a bond stretching vibrational mode that induces in-plane angle bending as well. As such, the dominant responsibility for the proper description of the temperature dependence lies with the anharmonicity of radial and in-plane angular terms of the potentials that is triggered as the atomic displacements increase with temperature. A wide temperature range was chosen, specifically *T* = 60 K, 160 K, 300 K, 500 K, 700 K, 1000 K, and 1500 K. At any given temperature and for each potential the lattice constant was pre-determined by performing multiple NPT simulations. These simulations were performed until the average pressure on either side of the cell was less than 10^−4^ of the maximum pressure fluctuations, at which point an average lattice constant was calculated. A 20 × 20 triclinic cell was constructed using the average lattice constant which was retained unaltered throughout all stages of the data acquisition simulations, i.e. velocity randomisation, temperature equilibration (NVT), and NVE simulations. This procedure wards off the possibility of the computational cell having unfavourable dimensions immediately before the primary simulation due to random pressure variation. Details on the values of the lattice constants obtained by the Tersoff-2010, AIREBO, and LCBOP potentials both by static relaxation as well as at finite temperature are given in the Supplementary Information. For each temperature 10 realisations were produced from which an average velocity autocorrelation sequence was computed. In Fig. 3 we have plotted the results obtained for all three potentials. It is immediately apparent that only the Tersoff-2010 potential properly produces a linear softening of the Γ-E_2g_ mode frequency. LCBOP gives variations in a large frequency range. The AIREBO and LCBOP potentials produce a non-linear non-monotonic behaviour for the Γ-E_2g_ temperature dependence. The Γ-E_2g_ frequency increases for temperatures up to around *T* = 500 K for AIREBO and *T* = 700 K–1000 K for LCBOP. This behaviour is not in qualitative accordance to available experimental results. At higher temperatures a quasi-linear behaviour is noted for both potentials with slopes of *χ* = –0.03 cm^−1^/K and *χ* = –0.06 cm^−1^/K for AIREBO and LCBOP, respectively. The first-order temperature coefficient obtained from the Tersoff-2010 potential is *χ* = –0.0517(9) cm^−1^/K, which is higher than the average experimental value by a factor of 2–3.

**Figure 4:**
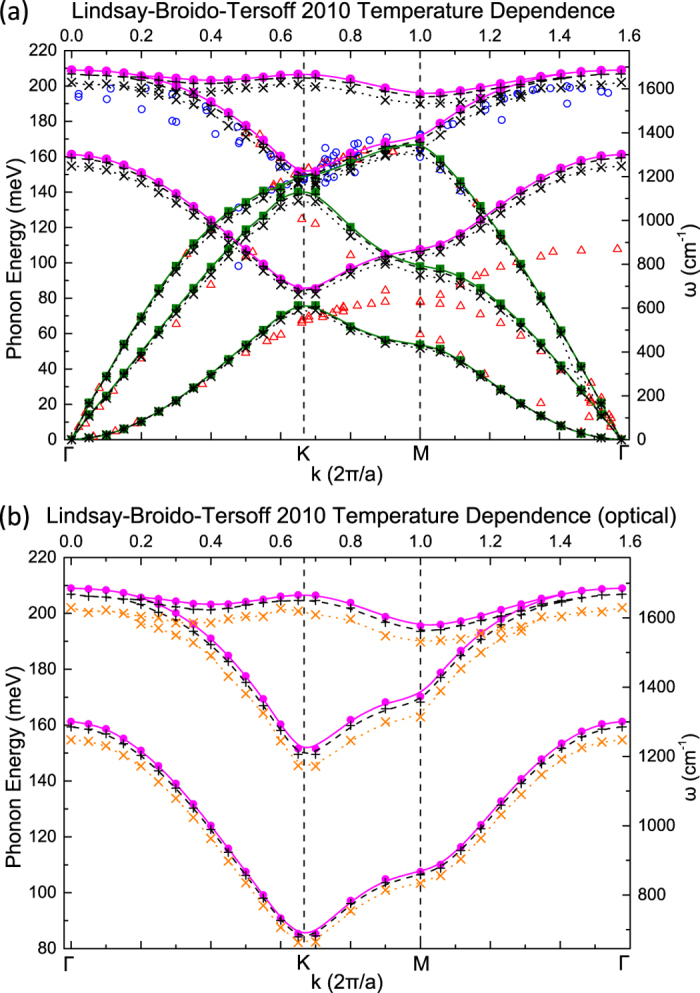
Temperature dependence of graphene’s phonon dispersion curves for the Tersoff-2010 potential. Data points for temperatures *T* = 60 K, *T* = 500 K and *T* = 1500 K are denoted by solid symbols, crosses and ×-marks, respectively. Plot (**b**) focuses on the optical branches, for which the temperature dependence is most notable. Open symbols in (**a**) correspond to experimental values taken from Refs 30,31.

To further examine the effects of temperature on the vibrational response of graphene we calculated the dispersion curves using the Tersoff-2010 potential for temperatures *T* = 60 K, 500 K, and 1500 K, which we show in Fig. 4. We note that the melting point of graphene employing this potential is around 2100 K. A stronger temperature dependence is observed on the optical branches. In Fig. 4b we focus on the optical branches, where it can be seen that upon increase of temperature the frequencies soften by as much as 75 cm^−1^ in this temperature range. The acoustic branches in the vicinity of Γ remain unaffected by changes in temperature. In the vicinity of the high symmetry points K and M a decrease in the frequencies of the acoustic branches is noted at most by 40 cm^−1^ in the considered temperature range.

## Conclusions

We have calculated the phonon dispersion curves of graphene by implementing and employing a method to compute phonon dispersion relations that makes use of atomic trajectories and velocities extracted from molecular dynamics simulations. We have suitably adapted the procedure to ensure the required spectral resolution to clearly obtain the six dispersion curves of graphene. From the potentials under study, the best overall description of the phonon dispersion curves, compared to experiment, were calculated using LCBOP, which also gives the most accurate TA, LO and TO branches. Tersoff-2010 produces the most accurate LA, and ZA, with the TO branch near to the accuracy of LCBOP. The original Tersoff potential surprisingly provides the most accurate ZO branch but fails dramatically on the highest optical branches. The reparameterised (by Lindsay and Broido) Tersoff-2010 potential produces dispersion curves of good accuracy compared to experiment, except for the ZO branch, and it is also the only potential that reproduces the correct linear temperature dependence of the Γ-E_2g_ vibrational mode, with a first-order temperature coefficient of *χ* = –0.05 cm^−1^/K. The temperature dependence of the dispersion curves was also calculated for the Tersoff-2010 potential, for a temperature range of *T* = 60 K–1500 K.

Although there are many theoretical works discussing phonon properties of graphene, there are only very few dealing with temperature effects^44^. Here we have presented numerical estimates of the temperature dependence of the G-peak in graphene. Although this has been experimentally explored in several studies, corresponding theoretical works are lacking. The method that we have used is generic in terms of applicability at different levels of theory, as long as the trajectories and velocities become available for a suitable simulation time.

In this work we have investigated the temperature dependence by employing well known empirical potentials widely used in the literature. We note that the Tersoff-2010^27^ correctly describes the linear *T*-dependence of the G-peak, with only a small quantitative discrepancy of the slope as compared to the experimentally available values. This does not stand for the LCBOP and AIREBO potentials, which are not in qualitative accordance to experiment, even though they sufficiently describe other properties of graphene. It is expected that force fields specifically designed for graphene^45^,^46^ may better succeed in reproducing the experimental data.

## Additional Information

**How to cite this article**: Koukaras, E. N. *et al.* Phonon properties of graphene derived from molecular dynamics simulations. *Sci. Rep.* **5**, 12923; doi: 10.1038/srep12923 (2015).

## Supplementary Material

Supplementary Information

## Figures and Tables

**Table 1:** Mean signed errors (MSE), mean absolute errors (MAE) and root-mean-square deviations (RMSD) of calculated points along dispersion curves with respect to experimental values, at *T* = 300 K.

PhononBranch	**Tersoff**			**Tersoff-2010**			**LCBOP**			**AIREBO**		
	**MSE**	**MAE**	**RMSD**	**MSE**	**MAE**	**RMSD**	**MSE**	**MAE**	**RMSD**	**MSE**	**MAE**	**RMSD**
LO	772.2	772.2	773.1	23.9	29.0	36.1	−20.3	28.5	32.8	114.3	114.3	123.4
TO	441.8	441.8	479.7	278.7	278.7	294.7	245.0	245.0	264.3	406.4	406.4	415.6
ZO	−14.1	32.7	37.5	222.8	222.8	238.8	−96.0	96.0	97.8	−174.2	174.2	185.4
LA	95.8	97.0	137.6	5.2	15.1	21.3	−25.2	26.2	32.4	33.1	37.0	47.1
TA	230.5	230.8	325.5	54.6	57.9	80.2	3.0	19.6	26.0	22.0	32.5	44.0
ZA	−55.1	55.1	71.5	7.7	26.1	38.8	−78.5	78.5	99.9	−70.8	70.8	92.3
Overall	329.7	347.7	472.2	88.8	94.4	149.8	9.2	74.8	118.1	76.5	136.9	193.7

Values are in cm^−1^
