# Supplementary material for: Phonon properties of graphene derived from molecular dynamics simulations
Source: Sci Rep. 2015 Aug 28;5:12923. doi: 10.1038/srep12923 (PMC4551956; doi:10.1038/srep12923)
Supplement: Supplementary Information [file srep12923-s1.doc]

Supplementary Information

Phonon properties of graphene derived from molecular dynamics simulations

Emmanuel N. Koukaras, George Kalosakas, Costas Galiotis, and Konstantinos Papagelis

## 1. Dispersion curves at *T* = 60K

**Figure SI-1.** Phonon dispersion curves of graphene calculated using (a) the LCBOP, (b) the AIREBO, (c) the original Tersoff (1989), and (d) the reparameterised Tersoff-2010 potential, at *T* = 60 K. Solid circles and squares correspond to numerical results of optical and acoustic branches, respectively. Open symbols correspond to experimental data taken from Refs. 2 and 3.

## 2. Comparisson of *k*VACS and Dynamical Matrix Diagonalization

We compare the dispersion curves obtained through diagonalization of the dynamical matrix with the ones obtained by the *k*VACS method described in the manuscript. For the comparison we have employed the Tersoff-2010 and original Tersoff potentials. The dispersion curves from diagonalization of the dynamical matrix are taken from the work of Lindsay & Broido [1] in which they construct and define the Tersoff-2010 potential. To be on equal footing the simulations were performed at a the low temperature of *T* = 60 K. At such low temperatures anharmonic effects are not expected to appear, thus we expect a good level of agreement between the two methods.

In figures **SI-2** and **SI-3** we have plotted the dispersion curves by both methods and for both potentials. The colored curves and points correspond to the method presented in the manuscript and the black dashed lines correspond to diagonalization of the dynamical matrix. As can easily be seen the agreement is by any standard *excellent*.

**Figure SI-2.** Comparison of the dispersion curves for graphene using the Tersoff-2010 potential, as obtained by diagonalization of the dynamical matrix (black dashed lines) and the *k*VACS method (coloured points and lines).

**Figure SI-3.** Comparison of the dispersion curves for graphene using the original Tersoff potential, as obtained by diagonalization of the dynamical matrix (black dashed lines) and the *k*VACS method (coloured points and lines).

## 3. Lattice constant temperature dependence

The lattice constant obtained by static relaxation from the original Tersoff, Tersoff-2010, AIREBO and LCBOP potentials are 2.53 Å, 2.49 Å, 2.42 Å, and 2.46 Å respectively. Compared to the value of at 2.47 Å reported by Mounet *et al.* [4], who have performed first principles calculations within the quasiharmonic approximation, the best value was produced using the LCBOP potential. In figure **SI-4** we show the temperature dependence for the lattice constant as obtained by the molecular dynamics simulations using the Tersoff-2010, AIREBO and LCBOP potentials. These are in very good agreement with the corresponding values reported in the work of Magnin *et al.* [5] obtained by classical Monte Carlo simulations in the isothermal–isobaric ensemble. From these potentials the most proper description of the lattice constant temperature dependence is by LCBOP, followed closely by AIREBO. As can be seen in figure **SI-4** the former produces a negative lattice expansion for graphene up to ~1000K and the latter up to ~450 K. On the other hand the Tersoff-2010 potential produces a positive thermal expansion throughout the temperature range examined. Mounet *et al.* find negative thermal expansion for graphene in the full range of temperatures that they examined (up to 2300 K). Experimentally the thermal expansion of monolayer and bilayer graphene has been studied by Yoon *et al.* [6] and found negative expansion coefficient for temperatures up to ~400 K.

**Figure SI-4.** Graphene lattice constant dependence on temperature calculated using the Tersoff-2010 (black squares), LCBOP (blue triangles) and AIREBO (red circles) potentials.

## 4. MSE, MAE and RMSD definitions

The mean signed errors (MSE), mean absolute errors (MAE), and root-mean-square deviations (RMSD) were calculated using the following equations.

$$\text{MSE}\left(y\right)=\frac{1}{N}\sum_{i=1}^{N} \left(y_{i}-Y_{i}\right)$$

$$\text{MAE}\left(y\right)=\frac{1}{N}\sum_{i=1}^{N} \left|y_{i}-Y_{i}\right|$$

$$\text{RMSD}\left(y\right)=\sqrt{\frac{1}{N}\sum_{i=1}^{N} \left(y_{i}-Y_{i}\right)^{2}}$$

where *y_i_* are the calculated values and *Y_i_* are the experimental values.

## References

[1] Lindsay, L. and Broido, D. A. Optimized Tersoff and Brenner empirical potential parameters for lattice dynamics and phonon thermal transport in carbon nanotubes and graphene. *Phys. Rev. B* **81**, 205441 (2010).

[2] Maultzsch, J., Reich, S., Thomsen, C., Requardt, H. & Ordejón, P. Phonon Dispersion in Graphite. *Phys. Rev. Lett.* **92**, 075501 (2004).

[3] Mohr, M., Maultzsch, J., Dobardžić, E., Reich, S., Milošević, I., Damnjanović, M., Bosak, A., Krisch, M., and Thomsen, C., Phonon dispersion of graphite by inelastic x-ray scattering. *Phys. Rev. B* **76**, 035439 (2007).

[4] Mounet N., and Marzari, N., First-principles determination of the structural, vibrational and thermodynamic properties of diamond, graphite, and derivatives. *Phys. Rev. B* **71**, 205214 (2005)

[5] Magnin, Y., Förster, G. D., Rabilloud, F., Calvo, F., Zappelli A., and Bichara C., Thermal expansion of free-standing graphene: benchmarking semi-empirical potentials. *J. Phys.: Condens. Matter* **26**, 185401 (2014)

[6] Yoon, D., Son, Y.-W., and Cheong, H., Negative Thermal Expansion Coefficient of Graphene Measured by Raman Spectroscopy. *Nano Lett.* **11**, 3227–3231, (2011)
